# Birth length and weight as predictors of breast cancer prognosis

**DOI:** 10.1186/1471-2407-10-115

**Published:** 2010-03-26

**Authors:** Bjørn O Mæhle, Lars J Vatten, Steinar Tretli

**Affiliations:** 1 Section of Pathology, the Gade Institute, University of Bergen, Bergen, Norway; 2 Department of Pathology, Haukeland University Hospital, Bergen, Norway; 3 Department of Public Health, Norwegian University of Science and Technology, Trondheim, Norway; 4 The Cancer Registry of Norway, Oslo, Norway

## Abstract

**Background:**

Birth size, and particularly birth length, is positively associated with breast cancer risk in adulthood. The objective of this study was to examine whether birth size is associated with survival among breast cancer patients.

**Methods:**

Information on birth size (weight, length and ponderal index (kg/length (m^3^)) was collected from birth archives for 331 breast cancer patients who were diagnosed at two university hospitals in Norway (Bergen and Trondheim). The patients were followed from the time of diagnosis until death from breast cancer, death from another cause, or to the end of follow-up, and birth size was related to survival, using Cox regression analysis.

**Results:**

Breast cancer patients with birth length ≥ 52 cm had nearly twice the risk of dying (hazard ratio, 1.92, 95% confidence interval, 1.09-3.41) from breast cancer compared to women with birth length less than 48 cm, after adjustment for place of birth and year of diagnosis.

Similar analyses related to birth weight and ponderal index showed no clear association with breast cancer survival.

**Conclusions:**

Poorer outcome of breast cancer patients with high birth length may reflect effects of factors that stimulate longitudinal growth and simultaneously increase the risk of metastases and fatal outcome. It is possible that the insulin-like growth factor (IGF) system is involved in the underlying mechanisms.

## Background

During the last two decades, it has been shown that birth size is positively associated with breast cancer risk later in life. Thus, birth weight, birth length, and head circumference have all shown positive associations with breast cancer risk in studies that employed measurements from birth records [1-3]. In a recent meta-analysis, it was suggested that birth length may be a better predictor than the other indicators of birth size [4], thus, the insulin like growth factor system (IGF) may somehow be implicated in the underlying mechanisms. Among factors measured in adulthood, it has been shown that body height is positively associated with breast cancer risk. High body mass index (BMI) is also associated with increased risk, but only among postmenopausal women. Among premenopausal women, BMI appears to be inversely related to breast cancer risk [5-7].

Less attention has been given to these factors as predictors of the prognosis among breast cancer patients. Nonetheless, it has been shown that adult obesity is adversely related to prognosis, both indicated as survival and mortality from breast cancer, and this effect appears to be equally strong for women with pre- and postmenopausal disease [8]. On the other hand, there is no clear evidence that body height is associated with survival from breast cancer [9]. With regard to birth size, we are not aware of any study that has assessed the relation with breast cancer survival.

In this study, we have therefore assessed whether birth size is associated with survival of subsequent breast cancer, and in the analysis, we have adjusted for the influence of known prognostic factors such as lymph node status and tumour diameter.

## Methods

In order to have access to data on birth size, eligible breast cancer patients had to be born in Trondheim or Bergen, Norway. Therefore, we used data from the Norwegian Cancer Registry to identify women with breast cancer who were residents in Trondheim or Bergen at the time of diagnosis. The Central Person Registry, which is administered by Statistics Norway, provided the necessary information on place of residence at birth. That registry maintains continuously updated records on each woman's residential address and the identity of the mother.

The information on birth weight and birth length for patients born in Trondheim was collected from the birth archive of EC Dahl's Foundation that served as the major birth clinic in Trondheim from 1910 to 1970. Birth data from Bergen were derived from archives of Bergen Birth Foundation and Midwife School, and later from the Maternity Hospital in Bergen. The information is stored at the Regional State Archives in Bergen. We further used information from the Central Person Registry of Norway to verify individual linkage between women of the study and their birth records.

To be included, patients had to be singletons born at term; birth length was restricted to range from 46 to 55 cm, and birth weight could range from 2000 to 5000 grams. In the analysis, we only included patients who were treated with modified radical mastectomy. Among 373 potentially eligible patients, we could include 331 patients in the analysis for whom we had sufficient information from birth. Perinatal information was abstracted from birth records and included birth weight (grams) and birth length (centimetres). As a measure of body mass, independent of length, ponderal index was calculated as birth weight divided by the cubed value of birth length (kg/length(m)^3^). The correlation coefficient between ponderal index and birth length was 0.00, and between ponderal index and birth weight it was 0.66, indicating that birth length and ponderal index may be considered independent of each other.

Breast cancer specimens were examined at the Department of Pathology, St Olav's University Hospital in Trondheim, or at the Department of Pathology, Haukeland University Hospital in Bergen. Information on tumour diameter and lymph node status at diagnosis was abstracted from the pathology reports.

In the statistical analysis, birth length, birth weight and ponderal index were categorised into approximate quintiles, and in subsequent analyses, the second, third and fourth quintile were combined into one category because of the range between the second and the fourth quintile was small. Thus, each variable had three categories: the first category was the lowest quintile; the second category was a combination of the second, third, and fourth quintile, and the third category was the highest quintile. For birth weight, the first quintile included weights below or equal to 3050 grams, and the fifth quintile included birth weights above 3850 grams. The corresponding threshold values for the lowest and highest quintile of birth length were 48 cm and 52 cm, and for ponderal index, it was 25.0 kg/m^3 ^and 29.1 kg/m^3^.

All patients were followed up by linkage to data from the Causes of Death Registry at Statistics Norway. Follow-up started at the date of diagnosis, and median follow-up was 108 months. Women were censored at the time of death, or at the end of follow-up. For patients in Trondheim, follow-up ended on December 31, 1999, and for patients in Bergen, follow-up ended on December 31, 2003. Death from breast cancer was the primary endpoint, and no patient was lost to follow-up. The Cox proportional hazards model was used to estimate the relative risk of dying (hazard ratio) from breast cancer related to the categories (see above) of birth weight, birth length and ponderal index. The Cox model is based on the assumption that death rates between categories of patients are constant over time. No serious deviations from the proportionality assumption were found, based on plots of the log minus log survival function (not shown). In a multivariable analysis, we adjusted for place of birth and year of diagnosis, and in additional analysis, we also took lymph node status and tumour diameter at diagnosis into account. All statistical analyses were done using SPSS, version 11.0 for Windows (SPSS Inc., Chicago, IL, USA).

The study was approved by the regional committee for ethics in medical research.

## Results

Patient characteristics are presented in Table 1, and Table 2 shows the associations of birth size indicators (length, weight and ponderal index) with breast cancer specific survival, after adjustment for place of birth and year of diagnosis. The results indicate that women who were longer than 52 cm at birth had 92% higher risk (hazard ratio 1.92, 95% confidence interval, 1.09-3.41) of dying from breast cancer compared to patients who were 48 cm or shorter. A test for linear trend across birth length provided a p-value of 3%. We found no clear associations with survival related to birth weight or ponderal index.

**Table 1:** Number of breast cancer patients and patient characteristics in Trondheim and in Bergen.

		Bergen			Trondheim	
	**No**	**Mean (SD)**	**Range**	**No**	**Mean (SD)**	**Range**

Age (years)	165	52.1(11.4)	27-83	166	49.0 (10.3)	30-73

Birth length (cm)	165	50.3 (1.62)	46-54	166	50.4 (1.91)	46-54

Birth weight (g)	164	3486 (466)	2310-4900	165	3436 (493)	2320-4800

Ponderal index (kg/m^3^)	164	27.3(2.69)	17.1-34.3	165	26.8 (2.2)	18.6-32.2

Positive lymph node status	138	39%		154	47%	

Tumour diam. (cm)	110	2.3 (1.3)	0.5-8.0	133	3.2 (1.9)	0-8.0

Number of breast cancer deaths	29			58		

Number of deaths from other causes	5			14		

Numbers lost to follow-up	0			0		

Follow-up time (months)	165	142	4-487	166	125	5-354

**Table 2:** Hazard ratios (with 95% confidence intervals) of dying from breast cancer according to birth length, birth weight and ponderal index, with adjustment for place of birth and year of diagnosis.

	Birth length	Birth weight	Ponderal index
	**N = 331**	**N = 329**	**N = 329**

1^st ^quintile	1.0 (reference)	1.0 (reference)	1.0 (reference)

2-4^th ^quintiles	1.08 (0.61,1.88)	1.08 (0.62,1.88)	1.01 (0.59,1.74)

5^th ^quintile	1.83 (1.03,3.25)	1.16 (0.59,2.29)	0.81 (0.39,1.67)

p-value linear trend	p = 0.03	p = 0.59	p = 0.11

Since ponderal index and birth length play independent roles, both variables were included in the subsequent multivariable analyses (Table 3). In addition, we included place of birth and year of diagnosis in the analysis (Analysis A, Table 3). The results were quite similar to those shown in Table 2. In subsequent analyses, we also included lymph node status (positive vs. negative) (Analysis B, Table 3), and tumour diameter (Analysis C, Table 3) as co-variates. Information on lymph node status was missing in 39 patients, and tumour diameter was missing in 86 patients; and in analysis B in Table 3, we therefore included 293 patients, and in analysis C, 246 patients were included. The results show that survival among patients within the highest quintile of birth length remained consistently poorer than for patients in lower categories of birth length, also after taking lymph node status and tumour diameter into account (Table 3). Detailed correlation analyses showed no correlations between birth length and tumour diameter (-0.056), and between birth length and lymph node status (-0.017).

**Table 3:** Hazard ratios (with 95% confidence intervals) of dying from breast cancer according to birth length and ponderal index.

	A	B	C
	**N = 329**	**N = 293**	**N = 246**

Birth length			

1^st ^quintile (< 48 cm)	1.0 (reference)	1.0 (reference)	1.0 (reference)

2-4^th ^quintiles (48-52 cm)	1.07 (0.61, 1.86)	1.15 (0.64, 2.08)	1.21 (0.61, 2.39)

5^th ^quintile (> 52 cm)	1.84 (1.03, 3.28)	1.88 (1.03, 3.45)	2.17 (1.06, 4.45)

Ponderal index			

1^st ^quintile	1.0 (reference)	1.0 (reference)	1.0 (reference)

2-4^th ^quintiles	0.93 (0.54, 1.61)	0.82 (0.47, 1.43)	0.95 (0.50, 1.83)

5^th ^quintile	0.80 (0.39, 1.65)	0.65 (0. 30, 1.38)	0.91 (0.38, 2.20)

Estimates adjusted for place of birth and year of diagnosis (A), with additional adjustment for lymph node status (B) and tumour diameter (C).

## Discussion and Conclusions

We have studied whether birth size is associated with the prognosis of breast cancer patients, and found that women with high birth length had poorer outcome than women with shorter length at birth. Specifically, women who were 52 cm or longer at birth had nearly twice the risk of dying from breast cancer compared to women who were shorter than 48 cm at birth. This association was not reduced after taking ponderal index, lymph node status, tumour diameter and place of birth into account. We found no association with survival related to birth weight or ponderal index.

In prognostic studies, predictors are usually assessed at the time of diagnosis, and patients are subsequently followed up in relation to outcome, which is typically time to death (survival) from the specified disease under study. In this study, however, measurements of birth size were made decades before the diagnosis (on average 50 years earlier). In the literature, there is no evidence that indicators of birth size have any influence on the prognosis of breast cancer once the diagnosis is established, and previously, no study has assessed survival of breast cancer related to birth size. However, given the magnitude of the association that we observed for birth length, sources of bias that may explain the finding should be carefully considered.

It is conceivable that better prognosis in one subgroup of patients could be due to better treatment quality. Birth length is positively correlated with adult height, and hence with socioeconomic status [10,11]. If quality of care and treatment followed a socio-demographic gradient, women with high birth length would be expected to receive better treatment. In that scenario the higher risk of dying from breast cancer related to high birth length that we found in our study is likely to be underestimated. In Norway, however, all cancer treatment is free of cost, and this is likely to reduce the potential for bias due to differences in access to and quality of treatment.

Another possible source of bias could be that the association of birth length with adult socioeconomic status, including higher education, could lead to earlier diagnosis in women with high birth length. This possibility could result in lead time bias, where survival from diagnosis to death would be longer among patients with high birth length compared to women with shorter birth length, who may be diagnosed at a later stage. Also under this scenario, however, the poorer outcome in patients with high birth length is likely to be underestimated.

The secular increase in birth length that took place in the 20^th ^century may also be a concern. In an attempt to take effects of calendar time (period) into account, we adjusted for year of birth, but this did not influence the estimates of effect. However, this adjustment may not be sufficient to account for secular changes in birth length.

It is also possible that important but unmeasured confounding factors could explain the poorer outcome of patients with high birth length. For such a factor to be of practical interest, however, it would have to be strong, and substantially influence breast cancer survival. Few known prognostic factors meet these criteria, and they are typically variables that describe the stage of the disease at the time of diagnosis, and therefore, these are not factors that could influence birth lenth. In the analysis, we adjusted for lymph node status and tumour diameter, but the adjustment did not substantially influence the results. However, body weight is a factor that does influence the prognosis of breast cancer patients [9]. Birth length is both associated with adult height and weight [11,12], but it is not clear if adult weight should be considered as a potentially confounding factor in relation to breast cancer survival. Maybe it is more appropriate to consider the effect of adult body size to be a possible mediator on the causal pathway from birth length to the outcome of breast cancer.

## Conclusions

It is difficult to see any practical implication of the finding that high birth length is associated with a poorer prognosis of breast cancer. Nonetheless, by establishing a link between conditions that operate *in utero *and the development and prognosis of later breast cancer, these results may be helpful in guiding investigators towards prognostic mechanisms. The relatively strong association of birth length, indicating longitudinal growth, may suggest that the insulin like growth factor (IGF) system is involved in the underlying mechanisms.

## Competing interests

The authors declare that they have no competing interests.

## Authors' contributions

BOM and LJV collected the data, and all three authors have contributed to the analysis of the data, interpretation of the findings, and to the writing of the manuscript. All authors have seen and approved the final manuscript.

## Pre-publication history

The pre-publication history for this paper can be accessed here:

http://www.biomedcentral.com/1471-2407/10/115/prepub
